# Case report: A rare case of acute myeloid leukemia with CPSF6–RARG fusion resembling acute promyelocytic leukemia

**DOI:** 10.3389/fonc.2022.1011023

**Published:** 2022-09-15

**Authors:** Junmei Zhao, Wentao Wang, Li Yan, Xi Chen, Wen Li, Wanying Li, Tingting Chen, Lunhua Chen

**Affiliations:** ^1^ Department of Hematology, Wuhan University Renmin Hospital, Wuhan, China; ^2^ Department of Infectious Diseases, Tongji Hospital, Tongji Medical College, Huazhong University of Science and Technology, Wuhan, China

**Keywords:** acute myeloid leukemia, CPSF6-RARG fusion, acute promyelocytic leukemia, resistance to ATRA, homoharringtonine

## Abstract

*Retinoic acid receptor gamma* (*RARG)* gene rearrangement has been reported in several acute myeloid leukemia (AML) patients. They resemble classical acute promyelocytic leukemia (APL) patients in clinical features, morphology, and immunophenotype but do not carry the *promyelocytic leukemia* (*PML*)*–RARA* fusion gene. Importantly, almost all these APL-like AML patients show resistance to all-trans retinoic acid (ATRA), and no effective treatment is recommended for them. Here, we identified a case of AML resembling APL in clinical presentation and experimental findings carrying a rare cleavage and polyadenylation-specific factor 6 (CPSF6)-RARG fusion gene. The patient was insensitive to ATRA and ATO but responded well to homoharringtonine and cytarabine.

## Introduction

Acute promyelocytic leukemia (APL) is a distinct subtype of acute myeloid leukemia (AML) characterized by abnormal accumulation of promyelocytes in bone marrow and coagulation abnormality. The hallmark of classic APL is the fusion gene and chimeric protein of promyelocytic leukemia and retinoic acid receptor-α (PML-RARA) caused by chromosome translocation t(15;17)(q24;q21) (1). PML-RARA oncoprotein inhibits the transcriptional activity of the *RARA* gene and disrupts the homeostatic function of PML, thus resulting in the proliferation of myeloid progenitors and maturation arrest at the promyelocytic stage (2). Importantly, the differentiation induction therapy with all-trans retinoic acid (ATRA) and arsenic trioxide (ATO) has strikingly improved the clinical outcome of classic APL patients. However, the *PML–RARA* fusion gene is absent in <2% APL patients, which is classified into variant APL or AML resembling APL (3). Among these, RARA rearrangement is relatively common with *PLZF-RARA* accounting for 50% variant APL. Retinoic acid receptor beta (RARB) and retinoic acid receptor gamma (RARG) rearrangements have also been demonstrated to generate AML resembling APL (4–6). The genetic heterogeneity, leukemogenesis mechanism, and optimal treatment regimen of these subtypes of AML remained to be elucidated, which pose a challenge to the recognition and treatment of variant APL. Herein, we identified a rare case of AML resembling APL with the *CPSF6-RARG* fusion gene who was resistant to ATRA and ATO but sensitive to homoharringtonine and cytarabine (HA) treatment.

## Case description

**Figure 1:**
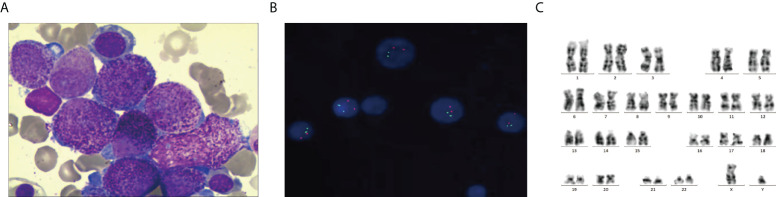
Morphology, FISH, and karyotyping analysis of the patient’s AML bone marrow (BM) sample. **(A)** Promyelocytes with hypergranulated cytoplasm and invaginated nuclei (Wright–Giemsa-stained BM smear, 1,000× magnification). **(B)** *PML–RARA* fusion signals were not detected by Interphase FISH using *PML– RARA* dual-color, dual-fusion translocation probes. **(C)** G-banded karyotype showing 46, XY [20].

The patient was a 28-year-old man with no significant past medical history who presented with a 1-week history of petechiae. His blood count showed a white blood cell (WBC) count of 29.21 × 10^9^/l, hemoglobin of 69 g/dl, and a platelet count of 103 × 10^9^/l. Fibrinogen and D-dimer levels were 2.99 g/l and 44.22 mg/ml, respectively. PT and APTT were 12 and 25.9 s, respectively. Bone marrow (BM) smear showed the hypercellularity with 41% predominantly abnormal hypergranular promyelocytes without Auer rods (**Figure 1A**). Cytochemical staining revealed that the abnormal promyelocytes had strong reactivity to myeloperoxidase (MPO). Flow cytometric immunophenotyping showed that the blasts were positive for CD13, CD33, CD117, CD38 (partial), and CD64 (partial) but negative for HLA-DR, CD34, CD14, CD56, CD7, CD10, CD5, CD2, CD3, CD4, CD8, CD19, CD20, and CD138. Thus, the diagnostic impression of APL was initially established.

However, multiplex RT-qPCR showed that all of the myeloid-related fusion transcripts were negative, including *PML-RARα*, *FIP1L1-RARα*, *PLZF-RARα*, *NPM-RARA*, *NUMA-RARα*, *STAT5-RARα*, and *PRKAR1A-RARα*. Fluorescence *in situ* hybridization also failed to detect the *PML-RARA* fusion gene (**Figure 1B**). Cytogenetic studies did not detect the translocation of t (15;17)(q24;q21) (**Figure 1C**). Whole-genome sequencing (WGS) identified *K-RAS* mutations in this patient.

The patient was immediately treated with all-trans retinoic acid (ATRA), and arsenic trioxide (ATO) was started in addition to ATRA on day 2. After 28 days of treatment, there were still 39% abnormal promyelocytes in BM, indicating that the patient was resistant to ATRA and ATO. Then, one course of DA regimen (DNR 60 mg/m^2^, d1–3, Ara-C 100 mg/m^2^, d1–7) was used as induction therapy. Unfortunately, there was still no response. The patient received HA chemotherapy regimen (homoharringtonine 4 mg/day, d1–7, Ara-C 100 mg/m^2^, d1–7) as reinduction therapy. The BM smear showed that the patient had achieved complete remission. Then, six courses of consolidation chemotherapy regimens were given as follows: 2 cycles of decitabine + CAG regimen (decitabine 20 mg/m^2^, d1–5; aclarubicin 10 mg/m^2^, d3–6; Ara-C 10 mg/m^2^/q12h, d3–14; G-CSF 200 μg/day until WBC count was >20 × 109/l), 2 cycles of the HA regimen (homoharringtonine 4 mg/day, d1–7, Ara-C 100 mg/m^2^, d1–7), and 2 cycles of the middle-dose cytarabine (2 g/m^2^/q12h d1–3). Up to now, the patient remains alive and was leukemia-free at follow-up.

**Figure 2:**
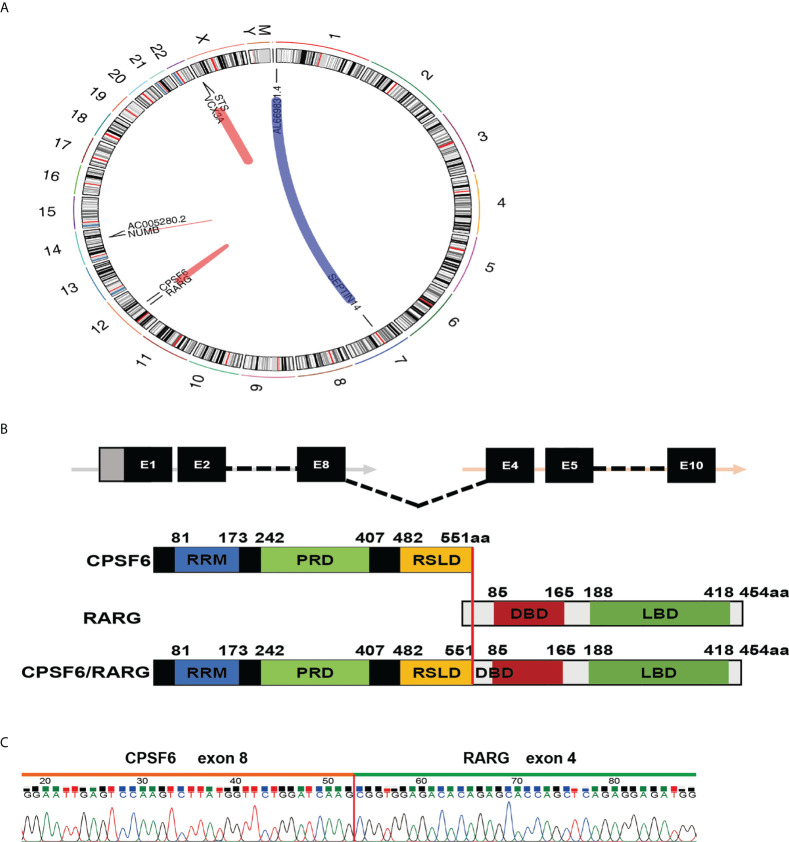
Molecular analysis of the CPSF6-RARG fusion. **(A)** Four fusion genes were found by analyzing RNA-sequencing data of the patient’s BM sample using deFuse inspector software. **(B)** Schematic diagram of *CPSF6, RARG, CPSF6–RARG* fusion transcript, and the fusion protein of the patient. The breakpoint is indicated by a red line. **(C)** Sanger sequencing of the PCR product analysis of the *CPSF6–RARG* fusion transcript junctions revealed a fusion between exon 8 of the *CPSF6* gene and exon 4 of the *RARG* gene.

In order to identify the driver fusion gene, transcriptome sequencing (RNA-seq) of bone marrow cells was performed. After analyzing the data, the patient was shown to have a rare fusion transcript within chromosome 12, in which the exon 8 of *CPSF6* was fused to the exon 4 of *RARG* (**Figures 2A, B**). To confirm the fusion, RT-PCR was performed using cDNA and the following pair of primers was used: forward (at *CPSF6* exon 8), 5′-AGATTGCCTTCATGGAATTG-3′ and reverse (at *RARG* exon 4), 5′-GGTAGCCAGAGGACTTGT-3′. The PCR product was detected and analyzed by electrophoresis and Sanger sequencing, respectively (**Figure 2C**).

## Discussion

Since 2018, six cases with *CPSF6-RARG* and one case with *RARG–CPSF6* have been reported (6–10). In previous studies, the fusing point was located at exon 4 of *CPSF6*; exon 1, exon 2, and exon 4 of *RARG*; exon 5 of *CPSF6*; exon 1 of *RARG*; intron 9 of *RARG*; and exon 6 of *CPSF6*, respectively. The present case provided the different transcript with *CPSF6* exon 8 fusing to *RARG* exon 4, further suggesting that the transcripts are highly varied.

All of the patients received ATRA therapy, but unfortunately, none showed any response to ATRA. In agreement with this, other RARG rearrangements also showed resistance to ATRA in clinic. Of note, *NUP98-RARG*-transformed murine HSPCs were sensitive to ATRA *ex vivo*. Additional chemotherapy drugs including cytarabine and/or anthracyclines were routinely recommended for high-risk APL patients in the course of induction therapy, but the regimen failed. Several patients received the salvage therapy after ineffective therapeutic regimen containing ATRA, and two patients showed a good treatment response. One patient achieved morphologic remission after a course of DA chemotherapy and received two courses of high-dose cytarabine therapy and two courses of standard 7 + 3 chemotherapy afterward and remained with complete remission until the last follow-up. Another patient underwent a course of HA treatment and achieved complete remission. In line with previous reports with *CPSF6-RARG*/*RARG-CPSF6*, the present case showed similar clinical manifestations, cell morphology, and immunophenotype with APL. The patient was resistant to combined treatment including ATRA, ATO, and DA chemotherapy but showed a very good response to HA chemotherapy.

Cleavage and polyadenylation specificity factor 6 (CPSF6) interacts with CPSF5 through its RNA recognition motif to enhance RNA binding and direct RNA looping. Breast cancer cells hijacked CPSF6 to promote A-to-I RNA editing for driving tumorigenesis and contributing to tumor heterogeneity (11). However, the role of CPSF6 in leukemia remained to be figured out. Another fusion protein harboring CPSF6 is CPSF6-FGFR1, which has been reported in a patient with myeloproliferative syndrome (12).

RARG consists of a DNA-binding domain (also named as retinoic acid response elements) and a ligand-binding domain (LBD), which share 90% homology with RARA and RARB. Unlike RARA-inducing granulocytic differentiation, RARG primarily maintains a balance between the self-renewal and differentiation of hematopoietic stem cells (HSCs) (12). Loss of RARG leads to the reduction in long-term repopulating HSCs but an increase in more committed hematopoietic progenitors in BM. Walkley et al. also found that RARG was very critical for maintaining a normal BM microenvironment, and *RARG^-/-^* mice displayed the abnormal accumulation of granulocyte/macrophage progenitors and granulocytes in bone marrow and peripheral blood, which rapidly developed into myeloproliferative syndrome (13). In addition to *CPSF6-RARG*/*RARG–CPSF6*, four other *RARG* rearrangements, namely, *NUP98-RARG*, *PML-RARG*, *NPM1-RARG-NPM1*, and *HNRNPC-RARG*, have also been identified in these AML-resembling patients. *In vitro* and *in vivo* studies had already demonstrated the oncogenic potential of *PML-RARG* (14). However, almost all of these AML subtypes with *RARG* gene rearrangements do not respond to treatment with ATRA and ATO and there is no unified expert consensus at present. Daunorubicin, idarubicin, homoharringtonine, and cytarabine as the induction chemotherapy regimen may work. Allogeneic hematopoietic stem cell transplantation (allo-HSCT) may be a preferred post-remission therapy in these special types of AML. Considering that the patient was very young, we have recommended allo-HSCT several times, but the patient refused due to economical load.

In addition to *CPSF6–RARG* fusion, *K-NAS* mutation was also identified by WGS in the present patient. *K-NAS* mutation leads to the continuous activation of Ras protein and may cooperate with CPSF6–RARG to participate in tumorigenesis (15). Among the previous reports (six cases with *CPSF6-RARG* and one case with *RARG–CPSF6*), *WT1* mutation occurred in four of seven patients, implying that the frequency of *WT1* mutation was higher than other mutation. *K-NAS* mutation was identified in two cases, and *DNMT3A*, *EZH2*, *NEAT1*, *BMPR1A* mutation was identified in one case, respectively. *WT1* plays an important role in hematopoiesis, and its mutation may influence the sensitivity to ATRA (16). *EZH2* is a histone H3K27 methyltransferase involved in epigenetic gene silencing (17). It has been reported that one patient exhibited an APL phenotype without a fusion gene but had *EZH2*-D185H mutation detected by targeted next-generation sequencing (18). The alteration of EZH2 function may be responsible for APL-like phenotype by dysregulation of the RARA and RARG genes. These additional gene mutations were also critical in the pathogenesis of APL and ATRA resistance. However, it is confusing that although *FLT3*-ITD is the most common mutation in APL patients, accounting for 35%, this mutation has not been found in *CPSF6–RARG* fusion AML patients so far.

In conclusion, we identified a case of AML resembling APL with CPSF6-RARG transcript who was resistant to ATRA and ATO but sensitive to HA treatment. Our findings imply that the HA regimen may be effective for patients with RARG rearrangements. Exploring the mechanism of ATRA resistance and finding more effective drugs are the research focus for the future.

## Data availability statement

The datasets presented in this article are not readily available because of ethical/privacy restrictions. Requests to access the datasets should be directed to the corresponding author.

## Ethics statement

This study was reviewed and approved by Renmin Hospital of Wuhan University Institutional Review Board. The patients/participants provided their written informed consent to participate in this study.

## Author contributions

JZ and WW designed the study, collected the material, analyzed the data, and wrote the manuscript. LY, WL, WYL, TC and XC collected the clinical samples and analyzed data. LC participated in analyzing the data and writing the manuscript. All authors contributed to the article and approved the submitted version.

## Funding

This work was supported by the grants from the National Natural Science Foundation of China (82000109 and 81900168).

## Conflict of interest

The authors declare that the research was conducted in the absence of any commercial or financial relationships that could be construed as a potential conflict of interest.

## Publisher’s note

All claims expressed in this article are solely those of the authors and do not necessarily represent those of their affiliated organizations, or those of the publisher, the editors and the reviewers. Any product that may be evaluated in this article, or claim that may be made by its manufacturer, is not guaranteed or endorsed by the publisher.
